# Preventing Fetal Alcohol Spectrum Disorder in Aboriginal Communities: A Methods Development Project

**DOI:** 10.1371/journal.pmed.0030008

**Published:** 2006-01-10

**Authors:** Paul Masotti, M. Anne George, Karen Szala-Meneok, A. Michel Morton, Christine Loock, Marilyn Van Bibber, Jennifer Ranford, Michael Fleming, Stuart MacLeod

## Abstract

The authors describe their three-year project working collaboratively with Aboriginal communities to prevent fetal alcohol spectrum disorder.

Fetal alcohol spectrum disorder (FASD) is an umbrella term that encompasses a spectrum of lifelong disabilities resulting from gestational (prenatal) alcohol exposure. At one end is the full fetal alcohol syndrome (FAS), involving craniofacial dysmorphology (particularly midfacial anomalies), growth retardation, and deficits in brain function. Less visible, but equally disabling, can be alcohol-related neurodevelopmental disorder (ARND), formerly referred to as fetal alcohol effects; children with this presentation along the spectrum tend to exhibit limited or absent dysmorphology but may have significant abnormalities in brain function, ranging from intellectual disability to more subtle but significant alterations in memory, judgment, and executive function [1].

Not surprisingly, FASD is more common in communities with high prevalence rates of drinking, such as some Canadian Aboriginal communities. Strategies to reduce alcohol use during pregnancy warrant local and national attention, but two barriers have impeded the development of successful strategies in Aboriginal communities. The first is the lack of resources and research capacity in some Aboriginal communities. The second is the historically grounded cynicism about research, in particular, university-based investigators conducting research *on* people and their communities.

Three questions informed our writing of this article. First, can community members, who may lack formal research training related to FASD, conduct health research and develop effective community interventions themselves? (Note that we are not suggesting that all communities lack research capacity and expertise in public health interventions. Many communities do possess great capacity and sophistication. However, we wanted to implement an approach that could serve communities without adequate resources.) Second, can a collaborative research agreement be reached between academic and Aboriginal communities that provides scientific support from academics and knowledge from community members, while ensuring autonomy and community ownership of the intervention? Third, most importantly, can such partnerships initiate interventions that effectively address pressing health issues? We think this is possible, as do four Aboriginal communities participating in a project funded by the Canadian Institutes of Health Research (CIHR)–Institute of Aboriginal Peoples' Health (IAPH).

This article describes a current three-year methods development project in which we, university-based researchers, are working collaboratively with Aboriginal communities interested in preventing FASD. The goal is for four independent communities to develop their own community-specific FASD interventions. We used a “participatory action research” (PAR) approach, which involves researchers studying a system and concurrently collaborating with members for positive social action. The use of PAR allowed the research team to address some of the historical problems associated with non-Aboriginal researchers conducting research in Aboriginal communities by capitalizing on the strengths of both parties [2]. We describe the research process, not the findings. Our goal is to encourage the use, evaluation, and refinement of our approach for a variety of public health issues.

## FASD as a Public Health Issue

FASD is an important public health issue that is preventable and underdiagnosed, and that has high human and financial costs [3]. It is the leading known preventable cause of intellectual disability in Western civilization [4,5]. North American prevalence rate estimates for full FAS range from 0.33–3 births per 1,000 in the general population [6,7], whereas the estimated rate for FASD is 9.1 per 1,000 [1,7]. Effects of FASD include primary disabilities such as physical malformations, growth restriction, permanent brain injury, learning disorders, maladaptive behaviors, and developmental disabilities. Secondary disabilities also occur and include higher rates of early school dropout, poorly recognized mental health conditions, and trouble with the law [8].

## Alcohol, FASD, and Aboriginal Communities

The Canadian Community Health Survey observed that 16.1% of women in the 15–44 age group, and 26% in the 20–24 age group, reported binge drinking 12 or more times in the previous year [9]. Square estimated that 16% of all pregnant women drink enough to be at risk for their fetus having alcohol-related birth defects [10]. In a Toronto study of pregnant women who sought counseling, 3.1% of clinic patients and 0.8% of women counseled by phone reported binge drinking during pregnancy [11]. In the United States, the Centers for Disease Control and Prevention reported similar findings, with 14.3% of nonpregnant women (18–44 years) engaging in at-risk drinking and 20% of pregnant women continuing to drink during pregnancy [5].

**Canadian Aboriginal People**

In Canada “Aboriginal” or “First Nations” is used to describe populations that would typically be described as “Native” or “American Indian” in the US or “Indigenous” in other countries. Canadian Aboriginal people and Native American persons share many common aspects of culture and history that are not separated by the US/Canada border. Similar public health issues and solutions exist across North American Native communities.

Aboriginal people have higher overall mortality and morbidity rates than non-Aboriginal people [12–16]. Some of this ill health may be attributed to alcohol abuse, which is the cause of FASD and which is consistently reported as a major problem by Aboriginal communities [12,14,17,18]. For example, May and Moran reported higher alcohol-related death rates for Aboriginal people than for non-Aboriginal populations [20].

Higher rates of binge drinking have been reported in Aboriginal communities in the US than in their non-Aboriginal counterparts [18–20]. A Québec study found that compared with the general population, fewer Aboriginal women were drinking; however, those who drank consumed greater quantities per occasion, with two-thirds drinking five or more drinks on a day that they consumed alcohol [21]. The Ontario First Nations Regional Population Health Survey indicated that significantly more Aboriginal females reported binge drinking than their counterparts in the general Canadian population [16]. Drinking during pregnancy is considered a public health concern, and binge drinking, possibly the most important risk factor for FASD, appears to be more common among Aboriginal women [21].

## Health Research in Aboriginal Communities

There are some excellent examples of successful community-based participatory action research in Aboriginal communities. However, in some instances, even when researchers adhered to strict standard research and ethical practice, the interests of Aboriginal communities were not protected [23].

Historically, research has been directed by university researchers from outside the community, with a clear delineation between researcher and community [2,22–31]. Today researchers are challenged to enter into more equal research partnerships. Aboriginal communities are also challenged to develop greater research capacities to address their own public health issues. PAR approaches often bring together the skills of formally trained researchers and indigenous people to both develop new knowledge and address health issues [2,22,24,26–28].

PAR, which is also referred to as community-based participatory research [32], begins with a different premise than standard research. “Participatory research begins with a problem” and emerges “as a way of empowering people to take effective action toward improving conditions in their lives” [24]. Communities identify a problem and devise the solution. Relationships between the various players in the research process differ from traditional research practice (Box 1).

**Box 1. PAR and the Relationships between Academic and Community Researchers**

**Green et al.**

“[PAR] seeks to link the processes of research, by which data are systematically collected and analyzed, with the purpose of taking action or affecting social change. To link the two processes, [PAR] demands a high level of participation by those most directly affected by the issue being studied” [28].

**Szala-Meneok and Lohfeld**

“There are many academic researchers or students who feel they are unable to work in the PAR model even though they believe in the principles underlying collaborative social science research in general, and in PAR specifically. We believe that even if a researcher cannot completely adhere to the principles of PAR, it is possible to conduct a modified project rather than following the more traditional style of social science research that is principal-investigator driven. In other words, it is possible for an academic researcher to apply several of the PAR principles, creating opportunities for community members to be research partners taking the lead in many aspects of conducting rather than serving as participants or sources of data….We think of social science research as existing along a continuum” [2].

**Kowalsky**

“…the research direction must come from the community. The focus of research then becomes the betterment of the community.…Researchers should be instrumental in the process rather than being in the centre of the process” [29].

**Gaventa**

“Participatory research attempts to break down the distinction between the researchers and the researched, the subjects and objects of knowledge production by participation of the people-for-themselves in the process of gaining and creating knowledge. In the process, research is seen not only as a process of creating knowledge, but simultaneously, as education and development of consciousness, and mobilization for action” [30].

**McAllister et al.**

“CBPR represents alternative orientations to inquiry that stress community partnership and action for social change and reductions in health inequities as integral parts of the research….CBPR is not a new research method but instead a new approach to public health research…the essence of the approach is a collaboration between researchers and community members, such that the expertise of each is shared to identify, study, and address health issues of importance to the community” [31].

While Aboriginal communities are undertaking more locally initiated research, many lack the resources and necessary research capacity to conduct research. University researchers may have this capacity but have inadequate understanding of the community's culture and health issues. Without such understanding, the researchers cannot identify appropriate research questions, which are better identified by community members themselves [2,27,28,31]. Academic researchers contribute technical knowledge about evidence and research methods, whereas community-based partners bring valuable indigenous knowledge about the background of the issue, the politics of the community, and what is feasible. Respecting and blending the contributions of both partners is vital to successful collaboration.

## The Healthy Communities, Mothers, and Children Project

This project (Figure 1) is funded by the CIHR–IAPH. The IAPH was established to “play a lead role in building research capacity in First Nations, Inuit and Métis communities.” [33] The intent of CIHR–IAPH is to “ensure that Aboriginal people are full partners in research, not merely informants and respondents.” [33]

**Figure 1:**
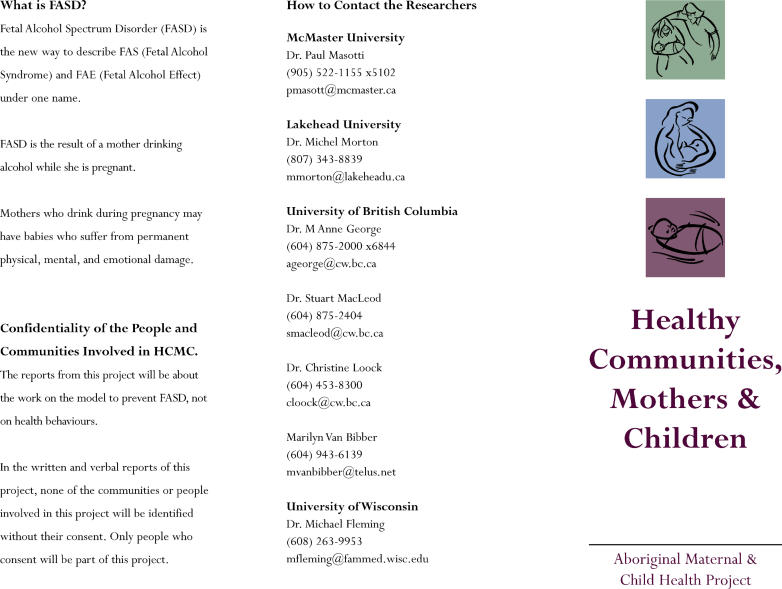
Brochure of the Healthy Communities, Mothers, and Children Project Image: Joanna Masotti

In drafting the research proposal, we had two main interests. One was to develop FASD preventive interventions that could work in Aboriginal communities. A second was to use research approaches that would be acceptable to Aboriginal communities in their efforts to address public health issues and build capacity. The result was a three-year collaborative effort. The rest of this article focuses on the first phase of this three-phase project that consists of a community-based participatory approach applied to the development of FASD interventions. Mutual and bidirectional capacity building has been vital to bringing together community members and academic researchers, and has contributed to the success of the research.

Eight researchers from four universities in Ontario, British Columbia, and Wisconsin developed the design for this multisite project. Five of the university-based researchers have worked, or have conducted research, with Aboriginal communities. One researcher is an Aboriginal person from the Yukon Territory. The university researchers asked decision makers in several Aboriginal communities to evaluate the proposal and to consider participating. While the general research questions were raised by the IAPH, the specific needs and research questions did not arise from the individual partner communities. As the research methodology was being developed, the collaborating communities were kept informed, but did not take the lead in refining the methods or the proposal. The ideal PAR approach would have featured a much earlier development of relationships between community and academic researchers, with the community-based people bringing forth the health issue.

In this project, the university researchers work collaboratively with four Aboriginal communities in Ontario and British Columbia, with two major aims. The first aim was to evolve working versions of the community-specific FASD interventions that were developed by each of the collaborating Aboriginal communities. The second aim was to describe, implement, and evaluate an FASD intervention development model for other communities interested in addressing FASD. There were five questions crucial to the project; we describe these questions below, and we also discuss the rationale used in reaching an agreement on the research approach implemented at the pilot sites.

## What Type of Intervention Should Be Used?

Key factors influencing our choice of intervention for FASD included effectiveness, cultural appropriateness, ability to meet a community's needs and characteristics, portability, and resources (e.g., training, personnel, people skills, equipment, and funding). In the end, we chose the “brief alcohol intervention” approach, which derives from motivational interviewing and cognitive behavioral therapy, and usually consists of one or more short counseling sessions that focus on changing behavior. In non-Aboriginal populations, the evidence indicates that brief alcohol interventions are effective, cost-effective, and versatile [33]. Success in reducing heavy alcohol use has been shown across various targeted behaviors, different populations, various delivery methods, and delivery by different interveners [34–40]. Brief alcohol interventions have also led to significant long-term reductions in alcohol consumption among women of childbearing age.

Typically, such interventions have five core characteristics: (1) identification of at-risk women, (2) assessment of drinking behaviors, (3) provision of information on the harmful effects of drinking, (4) method of delivery that facilitates decisions to adopt healthier drinking behaviors, and (5) monitoring of changes or progress [22]. To facilitate the initial stages of intervention development at each site, the Fleming brief alcohol intervention model was used as an example [36]. Although the Fleming model was designed to be an office-based “brief physician's advice” approach, we believe it provided an excellent example to study and build on.

## Would the Intervention Be More Effective in a Specific Target Population?

Initially, it was assumed that all women of childbearing age would constitute an appropriate target population for FASD prevention strategies. While this seemed self-evident, we had the opportunity of evaluating the possibility that the intervention might be more effective in a specific subgroup of these women. Women who had previously given birth were selected as the target population for the interventions, since evidence indicates that FASD is associated with increasing maternal age and parity [41]. Thus, the optimal time to identify at-risk women and prevent fetal injury may be after the first pregnancy. The results from an Ontario Federation of Indian Friendship Centres study support this approach, showing that more women reported drinking during a second pregnancy than during the first.

Targeting postpartum women addresses three important issues. First, women who are at highest risk for alcohol use during pregnancy may be the least likely to seek medical care early in pregnancy [43]. Mothers who are at risk for continued alcohol use can be identified through birth events such as postnatal checkups. Second, the intervention takes place prior to subsequent pregnancies, thereby preventing prenatal alcohol exposure [44]. Third, women who have recently given birth may have heightened interest in maternal and child health, which may benefit subsequent pregnancies.

## Do Setting Characteristics Affect the Development Process and Final Intervention?

Two urban and two rural communities agreed to participate in this project. The designation of urban or rural was based on several criteria that included community self-description. Possible differences between urban and rural settings included research capacity, available human/financial resources, levels of anonymity, and community support. Our goal was to improve the final intervention development model by adjusting for lessons learned from the unique experiences of the urban and rural partner sites.

## Which Elements of the Partnership Would Be Most Helpful?

A sense of “community ownership” was recognized as vital for the acceptance and effectiveness of the intervention. Our goal was to develop community research capacity. We wished to avoid obstructing community ownership, while still providing methodological expertise and enough, but not too much, consultative support. To best achieve these objectives, we (the university-based researchers) did not enter the communities and conduct research. Instead, community research facilitators (CRFs) were recruited from the communities, and continue to be responsible for facilitating and conducting all aspects of the research. The CRFs received training in leading workgroups. They were also provided with information on FASD, FASD interventions, and screening instruments. We have been in constant communication with and provide regular support to the CRFs.

This approach allowed much dialogue and a two-way transfer of knowledge, despite our off-site location. Our role was to provide access to information, methodological expertise, and a step-by-step process. The role of the community was, where appropriate, to make use of the suggested approach, request support when needed, document steps, and explain decisions. Moreover, it was agreed that community-specific data were owned by and were to remain in the community.

## Which Research Methodology Should We Use?

The nature of this partnership indicated that a PAR approach was an appropriate match for the roles of the partners that evolved over time. Initially, we were more active in guiding the project and the research procedures. As time passed, the communities' roles changed: we, the university-based team, began to act more like external consultants. This change made the project's approach to PAR different from standard applications. The project features three methodological phases: (1) community members developing interventions through a listening and learning process with local community people, (2) community members implementing and evaluating the interventions, and (3) both university-based researchers and community members documenting the process to provide a model for communities interested in developing and implementing similar interventions.

## Phase 1

### Local opinion leaders were identified

In a community survey of women of childbearing age, women were asked to identify people they know and trust, and who would be approached with questions on maternal and child health. The idea behind this approach was to help ensure that interventions were appropriate and acceptable to women. To accomplish this, we thought it was important for community leaders, who may not occupy formal positions of authority, to have the opportunity to participate in the development of a community-specific solution.

### Opinion leaders and CRFs developed the first version of the interventions

A unique community-specific intervention was developed at each of the four pilot sites. In each community, this was accomplished by organizing a work group involving up to ten local opinion leaders. Each workgroup analyzed its own data from a series of structured workgroup sessions designed to help develop the FASD intervention.

The workgroups were directed by a CRF, who received two types of support—training on workgroup methods and a structured approach to intervention and instrument development. The latter consisted of a manual composed of a series of written workgroup sessions that addressed specific components of the brief alcohol intervention and development process. For each workgroup session, goals and objectives were identified, and a series of questions was posed to assist in meeting specific objectives. In addition, relevant background information, such as examples of existing interventions or instruments, was provided. Table 1 illustrates the 12 workgroup sessions that were conducted as part of the structured approach.

**Table 1:**
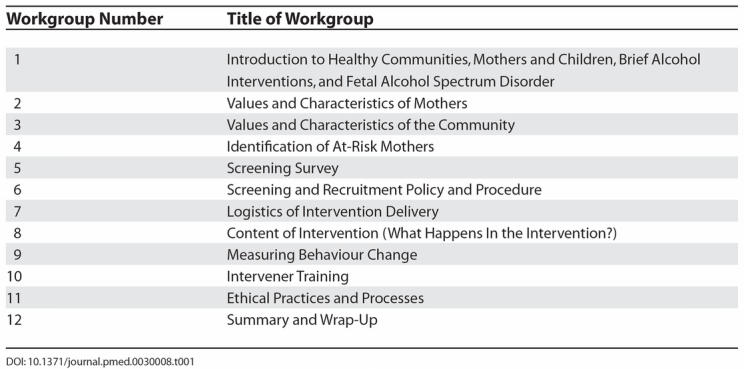
Workgroup Sessions: Community Research Facilitator and Popular Opinion Leaders

The structured approach to conducting the workgroups was presented and accepted as suggestions to each CRF to help with each component of the intervention development process. CRFs decided to use, modify, or ignore the suggested approach depending on the characteristics of the workgroup and the community's research capacity.

### A community advisory committee evaluated and helped with implementation

A method for providing oversight to the intervention development process was established. Each partner community agreed to implement a local community advisory committee responsible for operationalizing the intervention. These committees consisted of individuals who held positions of formal authority in their communities. At least one member of the workgroup and one of the university researchers could be invited to act in a consulting capacity. This second body provided a system of checks and balances regarding scientific merit and the economic and political feasibility of the interventions. Committee members included people from the local health department, the public health nurse or physician, and members of the Band Council or other governing bodies.

### A working version of the intervention was completed

The working interventions were developed at each site using a collaborative effort between the CRF, the community advisory committee, and the university researchers. Each included an overview of the intervention, the step-by-step procedures, the intervention content, the screening/data collection instruments, and the relevant background information.

## Phases 2 and 3

In phase 2, the four interventions will be pilot tested, with the main objective of conducting implementation analyses that will be used by each community to revise and improve the final versions. Specific questions to be addressed include the following: was the intervention implemented as planned? what were the unintended consequences or events? what components of the intervention worked well or did not work well? what were the characteristics of those who volunteered or declined to participate? how was the intervention viewed both by the participants and by those delivering it? what were the lessons learned? what should be changed or improved?

In phase 3, each community will draft a final working version of their community-specific intervention following revision decisions made after the implementation analyses. The university-based researchers will draft a final version of the overall Healthy Communities, Mothers, and Children Model.

## Discussion

At its core, we hope this study will offer significant findings that apply to Aboriginal communities seeking better health for their members and to university researchers pursuing a beneficial impact on public health.

A particular challenge for us as researchers was to determine an appropriate level of university support, given the differences among the four participating Aboriginal communities. A process of familiarization and development of trust was an essential antecedent to full engagement between both the community and the university partners. This process cannot be rushed. The main role of the communities was to make decisions based on the needs and characteristics of local women and communities, and to explain and document the rationale behind these decisions.

The approach we took recognizes mutual opportunities and strengths in the university–community partnership. Capacity building was viewed as an essential research product and as a two-way process. This project has given a clear indication that the community-based participatory research approach as implemented may be portable, that is, it can be used in other communities. In addition, it may be adaptable to address other public health issues such as smoking, obesity, diabetes, high cardiovascular risk, sudden infant death syndrome, substance abuse, and suicide.

### Next Steps

We intend to provide interested communities with two options. The first is access to each of the unique interventions. This includes manuals that clearly illustrate intervention content, tools, and procedures. This option could be chosen for communities that find one of the interventions to be an appropriate match for the community's needs/characteristics. The second is the “model,” which is a step-by-step guide for developing a community-specific intervention. The model will provide an overview of the project's PAR approach and will help build local capacity for communities that select this option.

To help make these options accessible to interested communities, a national advisory committee has been formed that is composed of Canadian and American experts in Aboriginal health, maternal and child health, health research, and FASD. The committee is supported by funding from Health Canada, and is responsible for guiding knowledge transfer activities and suggesting approaches to improving future collaborative research relationships between academic researchers and Aboriginal communities.

## Abbreviations

ARND: alcohol-related neurodevelopmental disorder
CIHR: Canadian Institutes of Health Research
CRF: community research facilitator
FAS: fetal alcohol syndrome
FASD: fetal alcohol spectrum disorder
IPAH: Institute of Aboriginal Peoples' Health
PAR: participatory action research
